# Dynamic graph exploration by interactively linked node-link diagrams and matrix visualizations

**DOI:** 10.1186/s42492-021-00088-8

**Published:** 2021-09-07

**Authors:** Michael Burch, Kiet Bennema ten Brinke, Adrien Castella, Ghassen Karray Sebastiaan Peters, Vasil Shteriyanov, Rinse Vlasvinkel

**Affiliations:** Eindhoven University of Technology, 5600MB Eindhoven, The Netherlands

**Keywords:** Dynamic graph visualization, Node-link diagrams, Adjacency matrices, Layouts, Reorderings

## Abstract

The visualization of dynamic graphs is a challenging task owing to the various properties of the underlying relational data and the additional time-varying property. For sparse and small graphs, the most efficient approach to such visualization is node-link diagrams, whereas for dense graphs with attached data, adjacency matrices might be the better choice. Because graphs can contain both properties, being globally sparse and locally dense, a combination of several visual metaphors as well as static and dynamic visualizations is beneficial. In this paper, a visually and algorithmically scalable approach that provides views and perspectives on graphs as interactively linked node-link and adjacency matrix visualizations is described. As the novelty of this technique, insights such as clusters or anomalies from one or several combined views can be used to influence the layout or reordering of the other views. Moreover, the importance of nodes and node groups can be detected, computed, and visualized by considering several layout and reordering properties in combination as well as different edge properties for the same set of nodes. As an additional feature set, an automatic identification of groups, clusters, and outliers is provided over time, and based on the visual outcome of the node-link and matrix visualizations, the repertoire of the supported layout and matrix reordering techniques is extended, and more interaction techniques are provided when considering the dynamics of the graph data. Finally, a small user experiment was conducted to investigate the usability of the proposed approach. The usefulness of the proposed tool is illustrated by applying it to a graph dataset, such as e co-authorships, co-citations, and a Comprehensible Perl Archive Network distribution.

## Introduction

Graph data, particularly dynamic graph data, occur in various fields of application such as call dependencies in software engineering [1], friendship relations in social networks [2–4], areas of interest connections in eye tracking data [5], or traffic situations in road networks [6].

**Fig. 1:**
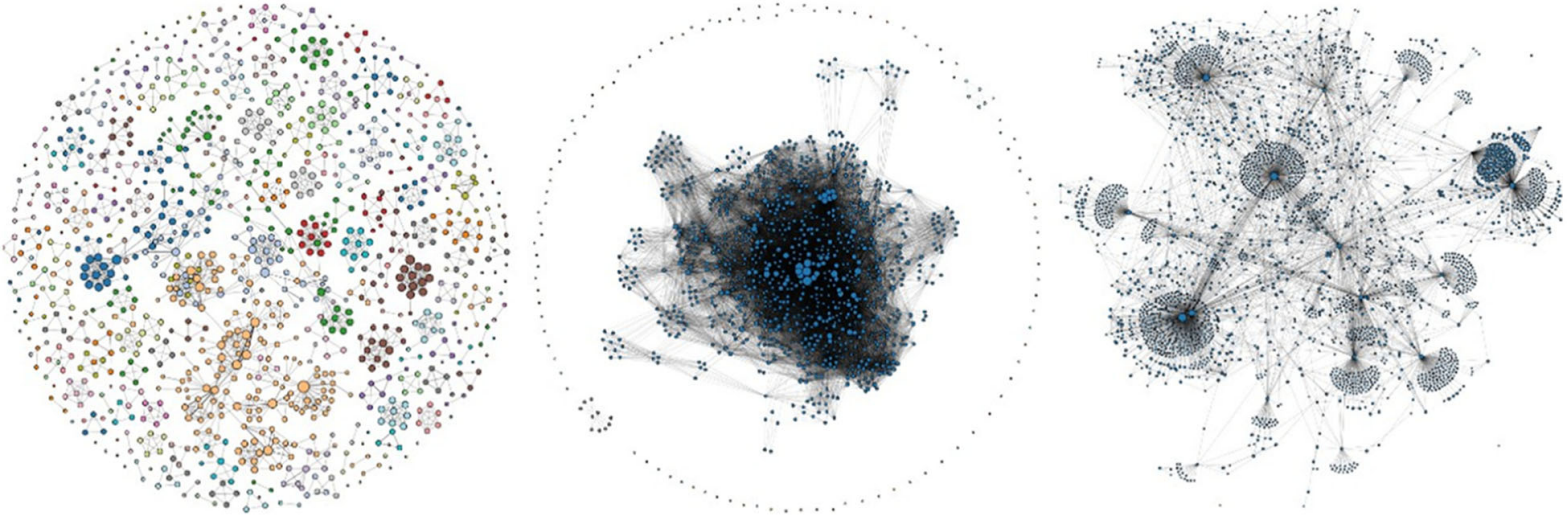
Node-link diagrams of real-world datasets: co-authorships, co-citations, and a Comprehensible Perl Archive Network (CPAN) distribution (cpa 2019) [16]

Exploring such data requires advanced visual metaphors, in the best case, interactively linking several of such metaphors to benefit from the positive effects of all of them [7]. For example, node-link diagrams [8] are useful for small and sparse graphs (see Fig. 1 for examples of already large and sometimes dense graph structures), whereas adjacency matrices [9] are best for large and dense networks [10, 11]. However, using only one concept may lead to a performance degradation in certain tasks [12].

To provide even more ways to find insight than just one fixed visualization technique, several node-link layouts are provided [13], along with several matrix reordering techniques [14]. The node-link diagrams follow esthetic graph drawing criteria [15] whereas the adjacency matrices support the finding of different grouping and clustering patterns depending on the user tasks and which reordering strategy is requested. Although all views and parameter adaptations can be selected upon user demand, the tool can also automatically suggest them based on graph data properties. The proposed novel linked visualization strategy provides ways to adapt a view based on insight from other views; for example, clusters found in an adjacency matrix can be used to guide the layout of the node-link diagram and vice versa.

The proposed novel technique allows an interactive selection of clusters in several views and allows the algorithm to compute the intersection of the node sets, a concept that is also extended to the dynamics of a graph structure. The output is a filtered version of a graph containing all nodes that occur for all the requested properties. This provides a clue regarding the importance of a node or node group based on algorithmic concepts such as layouts and reorderings, along with an additional enhancement through visual depictions of the output of such algorithms in combination, even for time-varying behaviors of selected node groups for certain visual features such as clusters and anomalies.

The novelty of this study in comparison to MatrixExplorer [2] can be described as follows:

### Visualization-based cluster and outlier detection

Clusters and groups of vertices can be automatically detected based on the visual properties given by the layout of a node-link diagram or the positions of the nodes in an adjacency matrix.

### Combined cluster information

Clusters or outliers can be identified in several views to link the identification processes for nodes that occur in clusters based on several layouts or matrix rearrangements. Two modes are supported: an intersection of common nodes and a union of all occurring nodes.

### Web-based

Providing web-based visualization makes it easier to start, without the need to install software or libraries. Moreover, along with the visual results, the data can be shared with other researchers through a dissemination process or even as a collaborative interaction.

### Scalability for vertices and edges

The application of a pixel-based representation supports the identification of node clusters and groups in extremely large graphs, although with matrix reordering, it can take quite some time until a suitable clustering result is generated.

This article is an invited extension of a formerly published conference paper [16] with special focus placed on the following aspects:

An extended repertoire of node-link layouts and matrix reordering techniques is provided (“Layouts and reorderings” section).

The automatic identification of groups, clusters, and outliers is supported, considering the dynamics of the graph data, based on the visual outcome as node-link diagrams and matrices (“Combining graph properties” section).

To visualize the dynamic graphs, views in the form of side-by-side node-link diagrams and adjacency matrices are provided as a type of time-to-space mapping [17] (“Dynamic graph visualizations” section).

Several more interaction techniques are implemented, as described in a list-based format (“Guidance-focused interaction techniques” section).

Finally, a small user evaluation is conducted to investigate the usability of the proposed interactive visualization tool (“User study” section).

## Related work

Graphs were initially introduced [18] to find a solution to the graph-theoretic problem, known as the “Seven Bridges of Königsberg”. To visually illustrate the problem, Euler used the visual metaphor of node-link diagrams [8]. Several years ago, they were powerful because the graph data were small and sparse, with only a few vertices and edges.

Currently, graphs are typically huge, and preprocessing and managing graph data is already a significant challenge [19]. However, visualizing relational data [20] as a node-link diagram is a naive approach leaving to visual clustering [12]. Even advanced layout algorithms [21] can only partially solve this problem because the immense size and density of the relational data do not allow the following individual outliers or anomalies. In most cases, these are occluded by dense graph regions, such as clusters of nodes.

Adjacency matrices are powerful visual concepts [9]. They allow thousands of vertices, and all weighted and directed edges in-between, to be represented. However, adjacency matrices show problems when following paths, which is also problematic for node-link diagrams if the graphs exceed a certain size [10, 22]. Moreover, an unordered adjacency matrix will not show any structure; hence, matrix reordering strategies, also guided by a human user [23] must be supported [14] Hybrid representations use the benefits of both concepts [3, 4]; however, a direct integration of both concepts does not support several independent layouts or a reordering, or at least makes them more difficult. By contrast, such a combination might hide insights that can be seen through node-link diagrams or adjacency matrices when represented separately. Consequently, Henry and Fekete’s [2] original approach is followed. Although their approach is already equipped with various features, the concept of exploiting visual properties from formerly laid out and reordered node-link diagrams and adjacency matrices is further added for node selection and filtering. If the graph data are not static, but dynamically change over time, visualization challenges occur [17]. However, apart from visualizing the dynamic graphs in several visual metaphors [7], it is challenging to compare the dynamics of the graphs from several perspectives [24]. Although this comparison can be conducted visually, it might also be supported by interaction techniques, allowing further analyses of the dynamic aspect in the graph data [25]. One possibility for visualizing time-varying graphs is by focusing on subgraphs to reduce the complexity of an analysis by using a supervised layout-based classification model [26]. Although this seems to be a powerful concept, an attempt was made to combine algorithmic, visual, and interactive approaches to build synergy effects for dynamic graph data exploration tasks. In addition, a pure algorithmic analysis, data transformation, and data storage and management play crucial roles in supporting the insights regarding this type of data [27]. However, regardless of how efficient an algorithmic solution is for a large dynamic graph, visual outputs based on a combination of visual metaphors are typically needed to guide the data exploration and analysis process [17] based on user decisions, as in a visual analytics system. This requires views and algorithmic processes on individual graphs, which create a dynamic graph if sequentially placed side-by-side.

However, including static and dynamic visual properties from corresponding graph visualizations and algorithmic processes have not been included in the modification or filtering process, targeting a better insight detection based on several visual features instead of only one. In this paper, focus is on displaying a graph in several views supporting the human observer by interactively changing the views, layouts, and reorderings on demand, inspired by a multiple visual metaphor approach [7, 28]. For this reason, insights from the views and parameter configurations are considered, along with the linking and highlighting of such insights.

## Data and visualizations

In this section, a data model for graphs is described, along with how a layout and reordering can be generated, which processes and algorithms are involved, and how visual graph properties can be combined and linked to influence other views and graph visualizations, particularly dynamic graph visualizations. Moreover, typical interaction techniques that are useful for guiding and navigating users in the created visualization tool are considered.

### Graph data

A graph *G* = (*V*, *E*) typically consists of a finite number of vertices *V* ≔ {*v*_1_,…, *v*_*n*_} and a finite number of edges *E* ≔ {*e*_1_, …, *e*_*m*_} ⊆ V × V. The edges might be weighted, meaning a weight function is attached, mapping each edge to a real-valued number, *f* : *E*_—→_*R*. In the special case of a dynamic graph, a model that describes the dynamics as a graph sequence consisting of individual static graphs can be used, as described above. These can be mathematically expressed as follows:

$$ \Gamma := \left\{{G}_1,\dots, {G}_k\right\}, $$

where *k* ∈ *N* models the number of graphs in this sequence. As an example, if a time component instead of a natural sequential order is involved, the indices can carry any number that models a time stamp.

### Layouts and reorderings

The layout of a graph *G* is the positioning of the given vertices *V* to *n* (normally) distinct locations in the display space, whereas the edges in between are drawn as either straight, curved, orthogonal [29], or partial links [30, 31], depending on several esthetic graph drawing criteria to be followed [32].

A reordering is applied to an adjacency matrix [14] following a certain property that produces a rearrangement of the vertices in such a way that a certain (typically user-defined) property holds, for example, a cluster structure at the diagonal.

For the layouts of node-link diagrams, the proposed repertoire is extended to support radial/circular, arc, force-directed, and hierarchical layouts. For the matrix reordering techniques, the guidelines given by ref. [14] are followed along with support reordering techniques from all described classes in their study. Moreover, a random-based approach is supported along with a reordering approach that considers the weight-based sum of the rows and columns. In the corresponding visualization tool, the user is able to select the node-link layout as well as the matrix reordering approach from the list of given options.

### Combining graph properties

As the novelty of this technique, insights such as clusters or anomalies from one view can be used to influence the layout or reordering of another view. The importance of nodes and node groups can be detected, computed, and visualized by considering several layouts and reordering properties in combination. Moreover, in the novel concept, users can take into account layouts and the reordering of several graph visualizations in a graph sequence, that is, the temporal information can also be considered to guide a dynamic graph exploration for relevant nodes or node groups. As the first step toward this direction, several node-link layouts and adjacency matrix reorderings of the same graph dataset are computed, and the visual properties of the produced diagrams are then considered to build an intersection or union set of the nodes under investigation. This principle helps identify certain node groups that belong together under different circumstances, meaning that their relations with each other are stronger than if only one representation is taken into account, which also holds for several graphs in a sequence, for example, making the appearance of node groups in an evolving cluster more relevant for an investigation.

The identification of clusters occurs in two stages: first, several algorithms are applied to a graph dataset, and second, the visual outputs of these algorithms are combined to detect strongly clustered nodes or nodes that do not fall in any of the clusters, which might be considered ‘real’ outliers. Such a feature is useful as a filtering function and has several benefits compared to the standard “one visualization” filtering.

Moreover, automatic node group detection can be applied based on the node group densities. This can be achieved for both a node-links layout and the adjacency matrices. Although this is a useful approach, it is best if human users equipped with the perceptual ability to rapidly detect visual patterns are involved in the node detection process [33].

Automatic computations of such relevant graph node properties, such as clusters, can be extended to dynamic graphs. For this reason, a static graph can be taken into account by defining a type of ground truth graph from which node groups are selected. This selection was then applied to all graphs from a sequence to depict their evolution over time. Furthermore, an algorithm can be allowed to identify stable parts in a dynamic graph, that is, identifying which clusters build the backbone of the evolution, indicating that they stay rather constant over time. These might provide a hint for strong node relations that persist over a longer time span than if the cluster oscillates around continuously. The goal of such an automatic computation based on several layouts and a matrix reordering is to provide an observer with additional views on stability patterns over time.

### Node-link diagrams and adjacency matrices

**Fig. 2:**
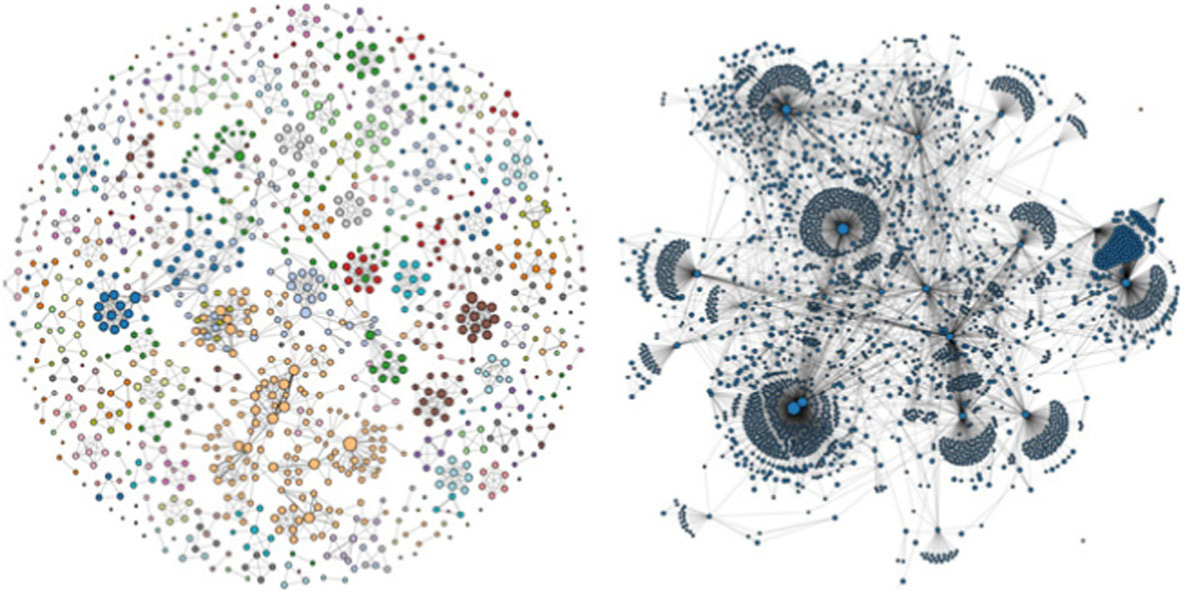
Left: Force-directed node-link diagram of a co-authorship dataset with 1053 nodes and 3504 edges; Right: Force-directed node-link diagram of the CPAN distribution dataset with 2724 nodes and 7669 edges [16]

Although the proposed original tool provided three layouts for a node-link diagram, i.e., force-directed [34], radial [35], and arc [6] layouts, the tool has been extended to support even more layouts such as a hierarchical layout. A force-directed layout presents the larger clusters close to the center and the smaller clusters and single isolated nodes close to the border of the diagram (Fig. 2). This helps to identify outliers better than if they are located inside a hairball-like node-link diagram.

**Fig. 3:**
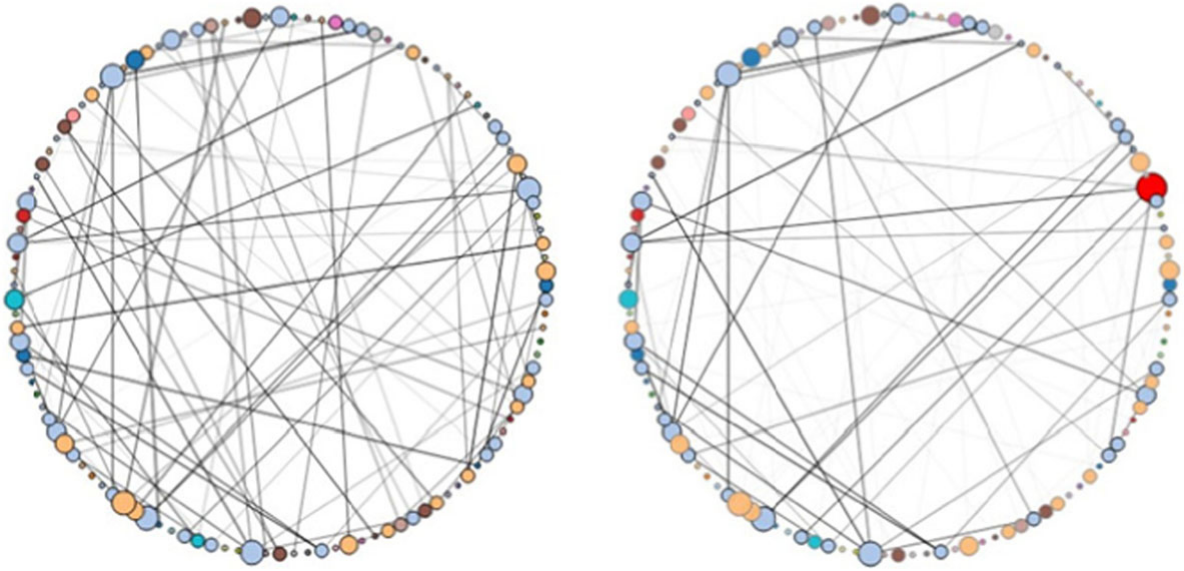
Radial layout of node-link diagram of randomly generated dataset. Left: Initial radial layout; Right: Filtered radial layout [16]

A radial layout displays the nodes aligned as a circle in which each node has an equal distance to the center, and edges are represented as straight lines connecting the nodes (Fig. 3). The radial layout is useful for determining the density of a graph in local regions and the connections between nodes.

**Fig. 4:**
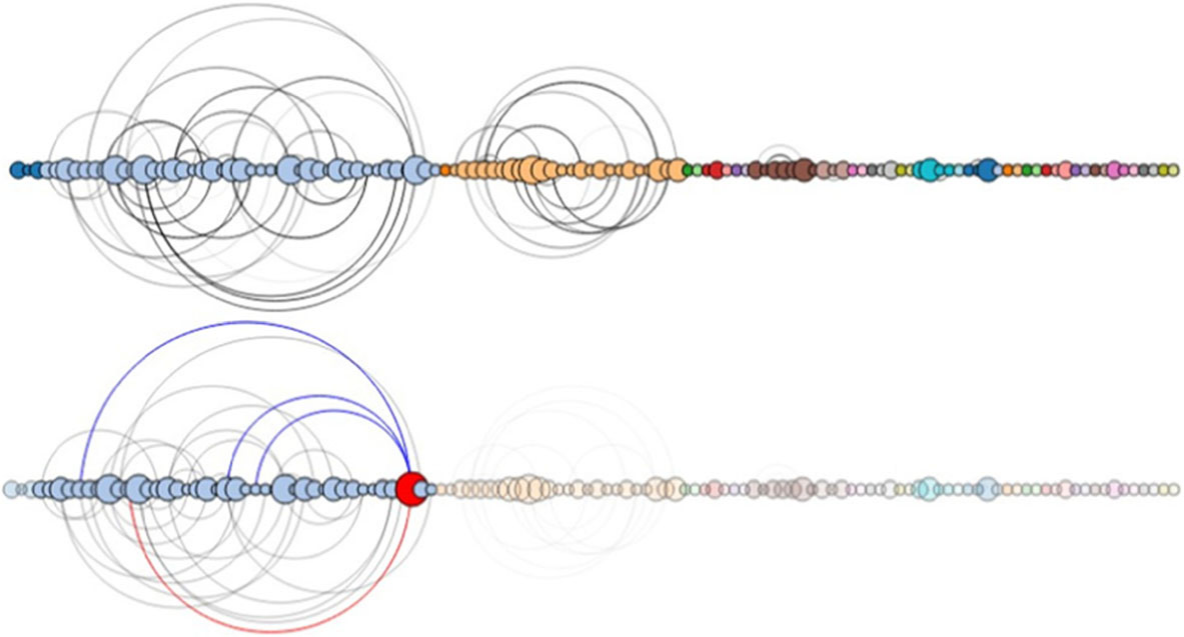
Arc layout of the node-link diagram of the randomly generated dataset. Top: The initial arc diagram; Bottom: Filtered arc diagram [16]

The arc layout aligns the nodes on a straight line, with edges represented as arcs connecting the nodes (Fig. 4). The arc layout has the advantage of being able to highlight the components if the node order is optimized.

**Fig. 5:**
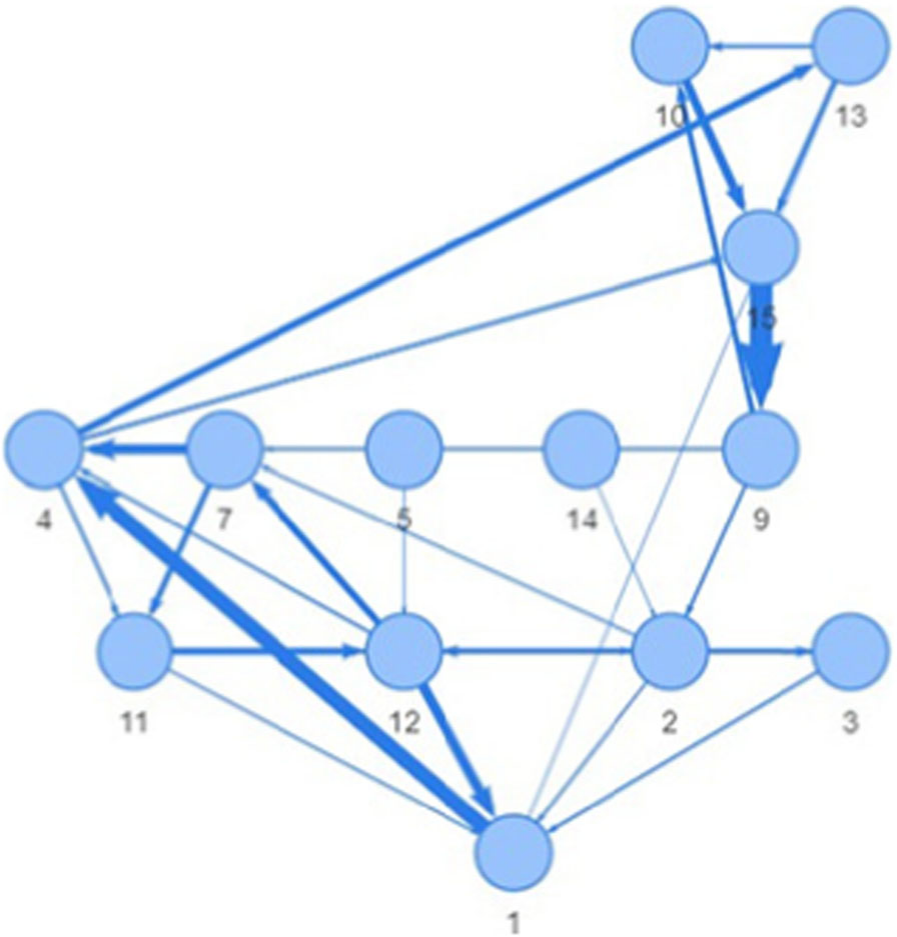
A hierarchical layout of a node-link diagram shows the graph nodes on horizontal layers

The hierarchical layout supports a flow in a graph, that is, the graph nodes are organized in horizontal layers with links connecting them (Fig. 5).

**Fig. 6:**
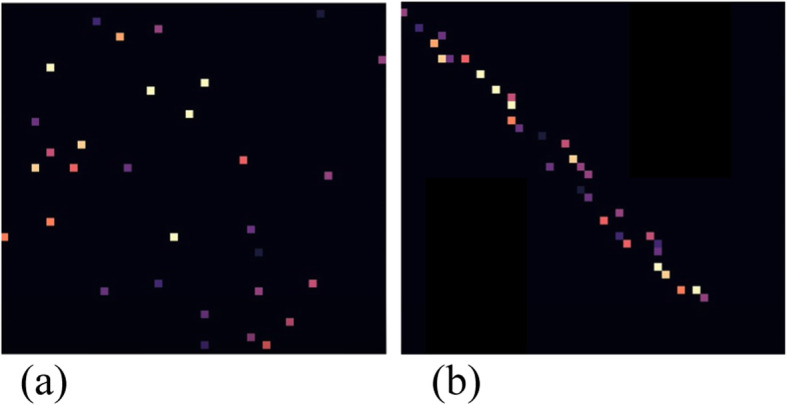
Illustration of a reordering: **a** Original adjacency matrix; **b** Reordered matrix based on a Cuthill–McKee order

Currently, the adjacency matrix is displayed as a pixel map, where the links are shown as filled grid cells where the corresponding connected nodes meet. The color of each cell depends on the weight of the edge, i.e., edges with a higher weight are lighter than edges with a lower weight (with the default color scheme). In the original tool, five reordering strategies are implemented, and the list is extended using all reordering strategies described in the study by ref. [14], as well as a random reordering and an option to return to the original ordering (see Fig. 6 for a reordering example in a simple adjacency matrix visualization), which is called a reverse Cuthill–McKee order. More orderings are possible, which are not mentioned above, such as a unique value order, a mean value order, and a random permutation order, inspired by the study conducted by ref. [14].

### Dynamic graph visualizations

**Fig. 7:**
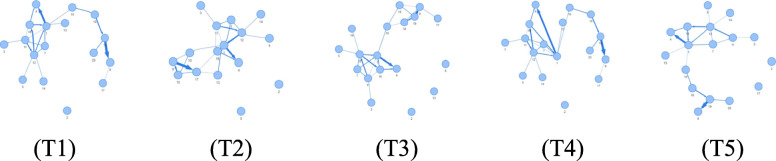
An illustrating example for a dynamic graph consisting of 5 time steps (T1) to (T5) shown in a node-link diagram in a force-directed layout. Simply inspecting the dynamic graph does not really help identify dynamic visual patterns, and hence algorithmic and visual concepts must be integrated to achieve the full potential of the approach

To support views and perspectives on dynamic graphs, side-by-side views called time-to-space mappings are provided [17]. In the present study, two major visual metaphors are followed for the individual static graphs in a sequence, i.e., node-link diagrams and adjacency matrices. The user can see both of them placed next to each other and can adapt a visual metaphor and the layout or reordering on demand, either for the entire sequence or each static graph in the sequence individually [7]. Figure 7 gives an impression of a sequence of static graphs in a force-directed layout from which individual node groups can be selected or automatically computed based on certain visual properties, for example, all lying on the same horizontal layer. The user is able to decide if those node groups are to be further inspected based on their union (all of them together) or their intersection (only the overlap), which is a stronger selection criterion than the union operation.

### Guidance-focused interaction techniques

**Fig. 8:**
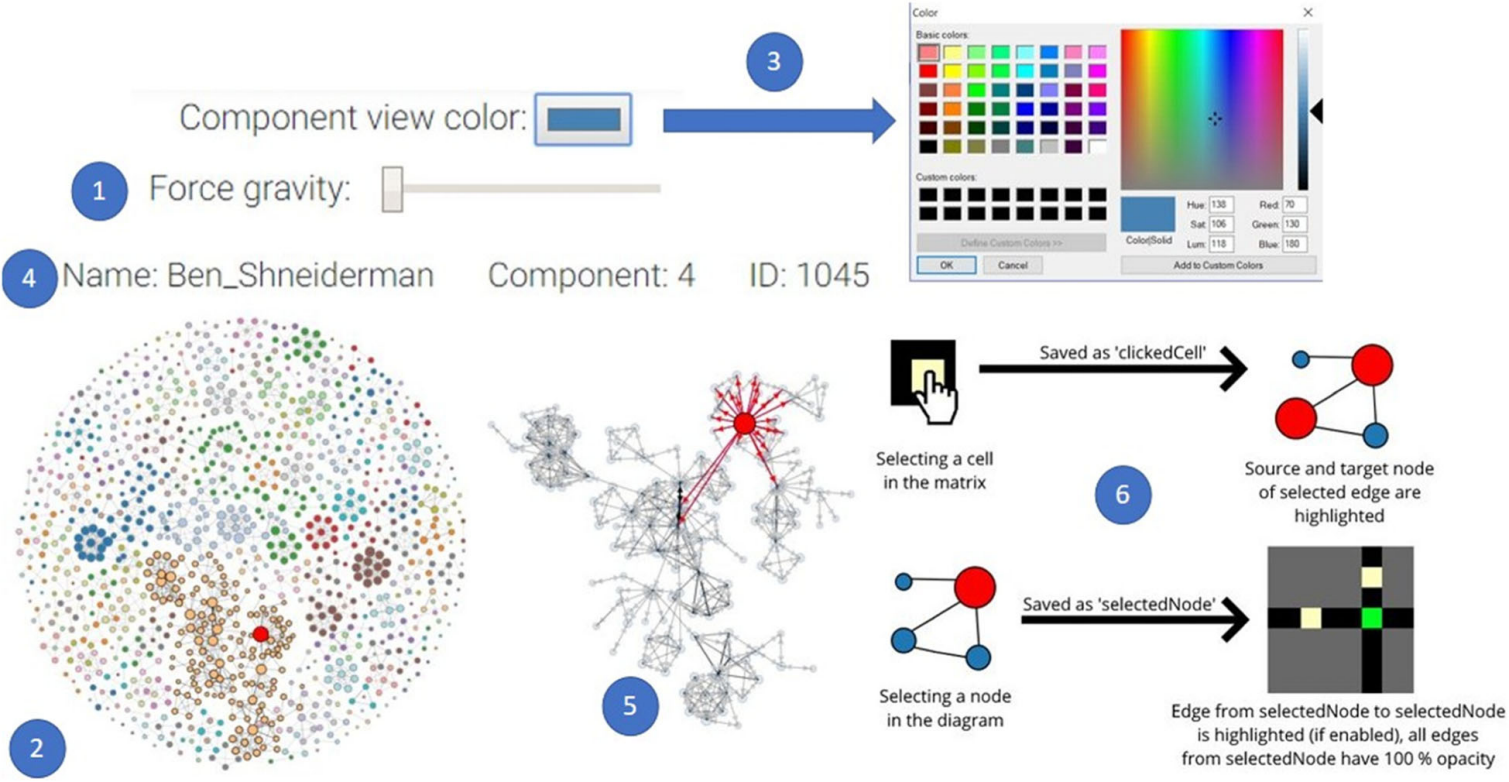
An illustration of several interaction principles included in the tool: A layout or matrix reordering of a graph can be generated and the corresponding visualization can then be shown. Selecting a node and computing the cluster it belongs to is a powerful concept; however, the user can also manually select nodes and node groups for further explorations

The proposed tool includes the following interactions (see Fig. 8 for some examples) based on the visualization interactions presented in ref. [36].

#### Select

**Fig. 9:**
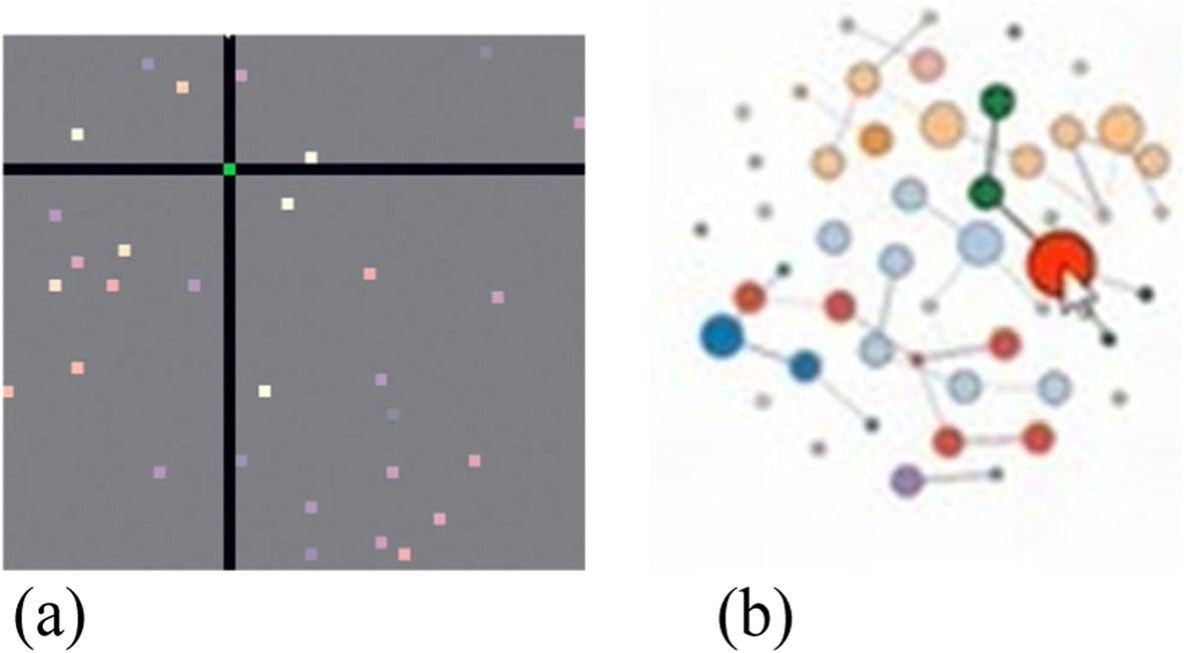
A node, a node group, an edge, or an edge group can be selected in a **a** matrix or **b** node-link diagram

When the user clicks a node in the node-link diagram, the node is selected and changes its color to red. Users can select nodes and edges in the adjacency matrix by clicking on the cells inside (see Fig. 9 for an illustrative example from an actual small dataset).

#### Explore

Users are offered panning and the ability to zoom in and out of the matrix. Panning works by clicking on the matrix and dragging it with the mouse. Zooming in and out can be achieved using the mouse wheel. The scalable vector graphics (SVG) view of the node-link diagram also allows users to zoom in and out with the mouse wheel. Another form of possible zooming is to change the distance of the nodes using a slider, which also changes the size of the graph.

#### Reconfigure

**Fig. 10:**
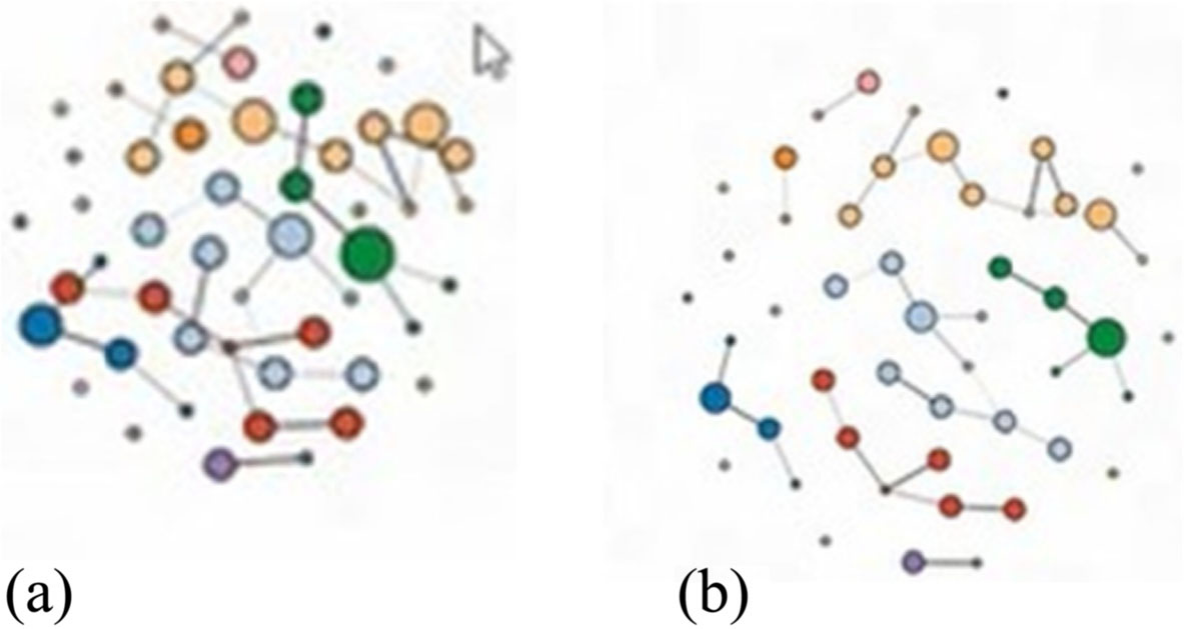
**a** A node-link diagram can be enhanced by applying **b** a force-directed algorithm to generate a better group- preserving layout

Each layout features a slider that rearranges the data based on the distance between nodes. For example, if a dataset was originally presented in a shrunken configuration, users can increase the distance between the nodes to better analyze the data. To implement this, the value set by the slider is being used to change the distance between nodes, the force gravity, or the circle radius in the functions that draw the node-link diagram. The original layout generation also belongs to this interaction category (Fig. 10).

#### Encode

The color nodes in the node-link diagram belonging to the same component with the same color. In this way, the user can easily differentiate between components. Users are also offered a button to change the hue, saturation, and brightness of the nodes in the SVG view. This can be achieved using the information regarding the components in the json file of the dataset and avaScript-based visualization library (D3) commands to set the color of the nodes.

#### Elaborate

When the users select a node in the node-link diagram, they can see the node’s ID, name, and component. If the users select an edge in the matrix, they can see information about its source, target, and weight. This is possible because the information can be extracted from the json file of the data for each node or edge using D3.

#### Filter

When the users select a component by clicking on a node belonging to it in the canvas view, an SVG view displays only this component next to the canvas view. In the SVG view, the user can see the incoming/outgoing edges displayed with arrowheads. A D3 example was used as an inspiration for the proposed SVG view. In addition, when users select nodes in the radial or arc layouts of the node-link diagram, the selected component is highlighted.

#### Connect

When users select an edge in the matrix, the corresponding nodes linked by this edge are colored red in the node-link diagram. If highlighting is enabled when users sect a node in the node-link diagram, the matrix will highlight all edges connected to this node. This is achieved using global variables that store information about the nodes and edges, and a select function that detects if a node or edge is selected. The information of these variables is then updated based on the selected object.

## Guiding graph exploration

The proposed tool offers many possibilities for inspecting a graph dataset and applying algorithmic processes. On a general level, the exploration between static and dynamic graphs can be distinguished.

### Exploring static graphs

The usefulness of the proposed visualization tool is illustrated by applying it to an author similarity dataset and show an adjacency matrix, with two datasets for the force-directed layout of the node-link diagram containing CPAN distributions and co-authorship data. A small generated dataset will also be used to showcase the radial and arc layout as a node-link diagram. Although different datasets will be used because adjacency matrices with a high number of edges are more interesting to study, the node-link diagram struggles with rendering graphs with a high number of edges, and the radial and arc layouts are more useful for smaller datasets.

**Fig. 11:**
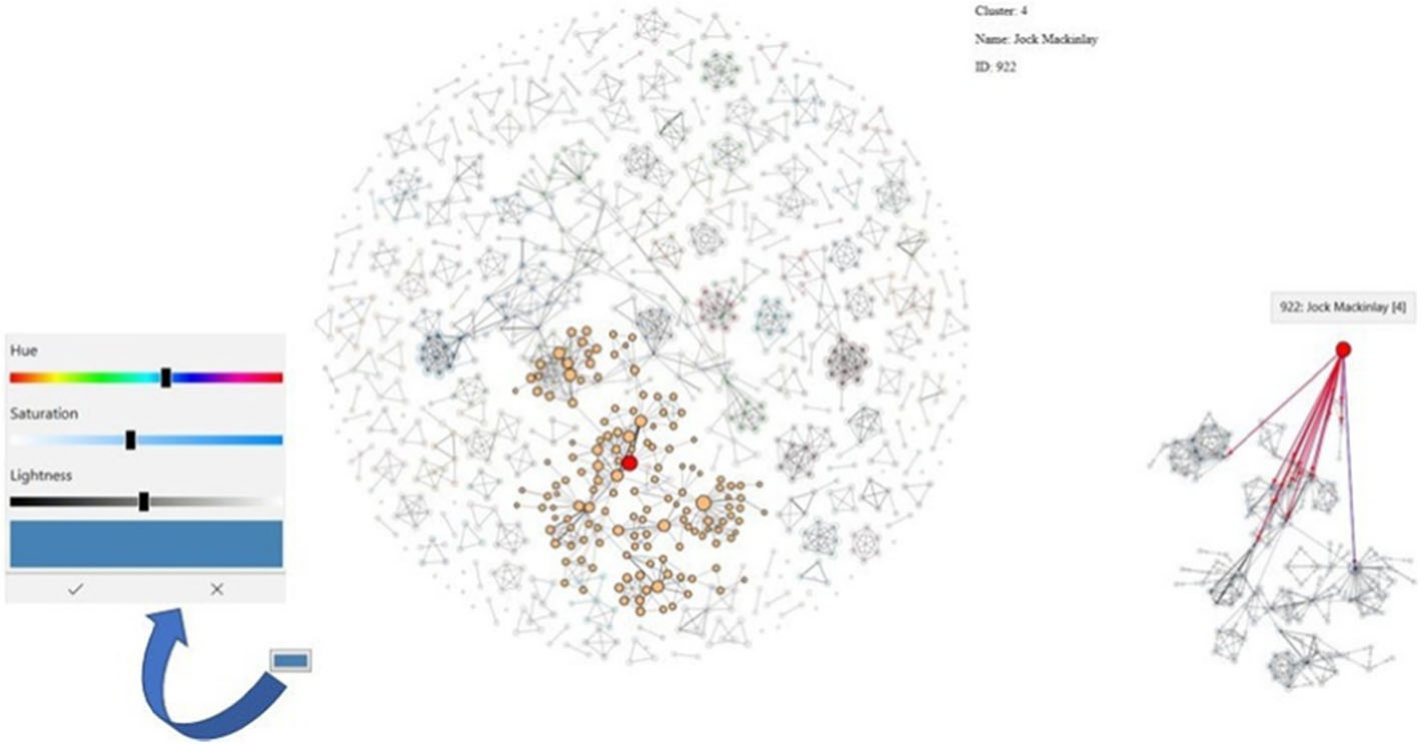
Selecting a cluster of nodes to take a closer look at the details, e.g., a node that is important in this cluster

The co-authorship data included 1053 nodes and 3504 edges. Once the users have uploaded the dataset, they can view it as a node-link diagram. The force-directed layout of the node-link diagram displaying the co-authorship dataset is shown on the left in Fig. 2. Users can see that larger connected components are displayed at the center of the visualization, whereas smaller components are displayed relatively closer to the border of the visualization, and single isolated nodes are even closer to the border. In addition, nodes with a larger number of incoming edges appear larger than the others. By using this information, it can be concluded that the single nodes can be considered as outliers and ignored; hence, they do not flow into the selection of the most important and strongly connected nodes. Finally, it can be observed that every connected component of the graph has its own color, making it easy for the users to differentiate between the different components of the graph. Selecting individual components from the node-link layouts can lead to useful insight that cannot be found by the standard solutions (Fig. 11).

The CPAN distribution dataset contained 2724 nodes and 7669 edges. The force-directed layout of the node-link diagram for this dataset is shown on the right side of Fig. 2. When the graph is rendered, users can note how most of the nodes in the graph are connected to a large component. Within it, there are a few nodes that appear larger, having a higher number of incoming edges. In addition, these nodes appear to be surrounded by groups of smaller nodes with fewer edges. These nodes represent packages upon which other packages depend. They are also more frequently used compared to the others. Nodes that are not part of the large connected component can be interpreted as outliers and are therefore ignored.

The randomly generated dataset contains 120 nodes, the maximum weight of an edge is 30, and the chance of an edge is 0.007 (0.7%). The radial layout for this dataset is shown in Fig. 3, and the arc layout is shown in Fig. 4. In Fig. 3, the initial radial layout on the left shows the entire dataset. In the filtered radial layout on the right, users can see that most of the edges belong to the component of blue nodes if they select a node from the component of blue nodes. The arc layout can help the user find co-occurrences in the data. In Fig. 4 (left), the initial layout reveals the connected components in the graph. The filtered layout on the right reveals the connections of a selected node within its component.

**Fig. 12:**
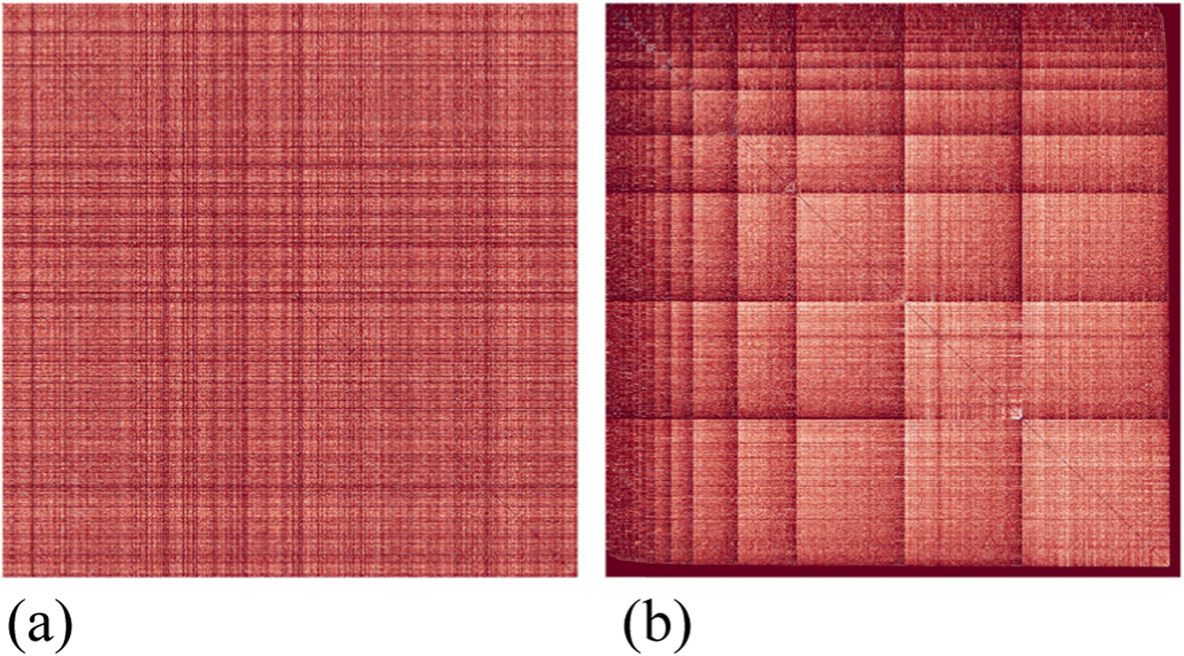
**a**: The adjacency matrix of the initial author-similarity dataset with 1053 nodes and 907164 edges; **b**: The reordered dataset using the reverse Cuthill-McKee algorithm [16]

The author-similarity data included 1053 nodes and 907164 edges. The initial adjacency matrix displaying this dataset is shown in Fig. 12 (a). In the initial order, no clear patterns were observed. However, when the reverse Cuthill–McKee ordering is executed on the data, users can see symmetric diagonal block patterns. The reordered matrix is shown in Fig. 12 (b).

**Fig. 13:**
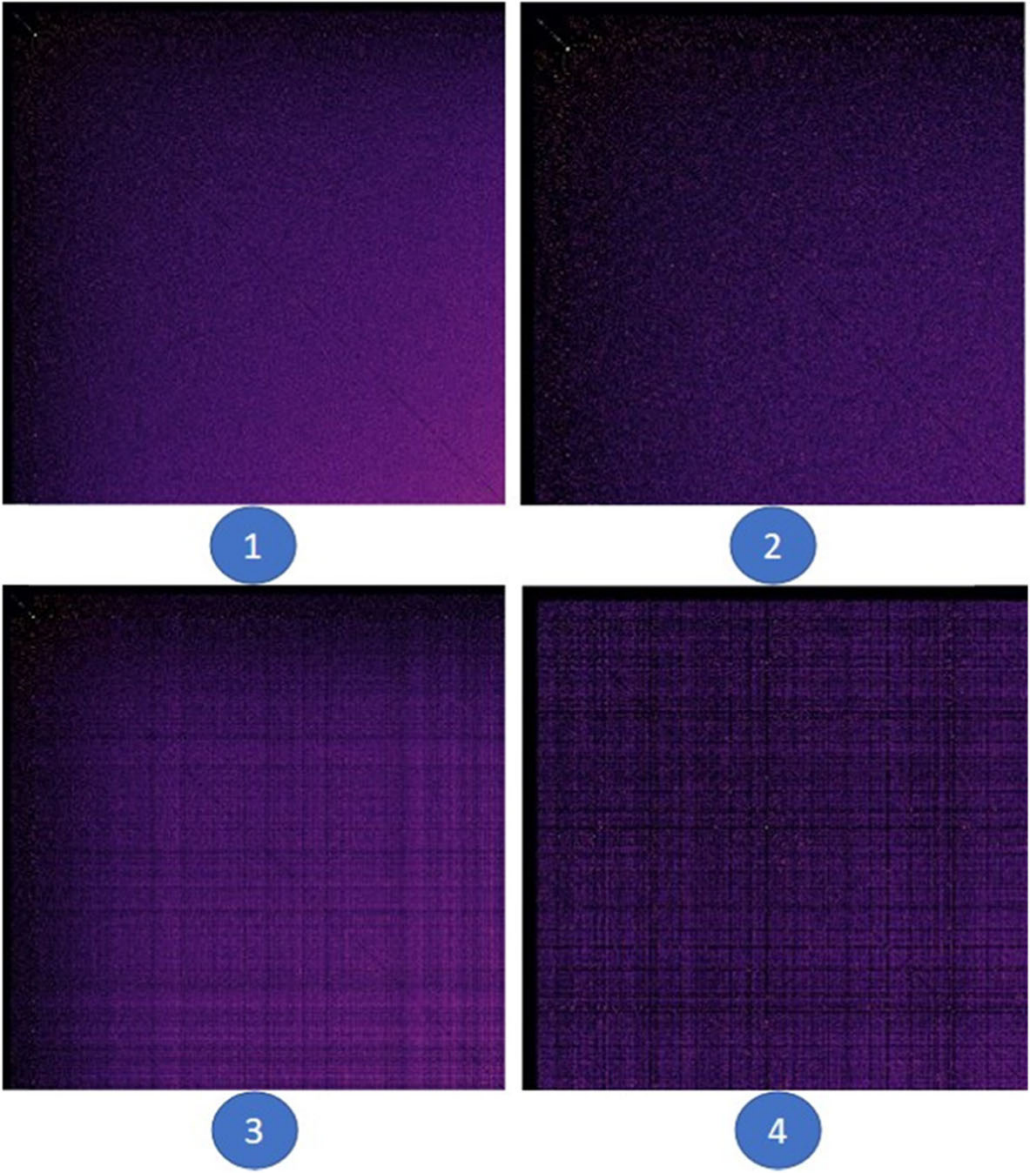
Matrices of the author-similarity dataset reordered using the (1) mean value ordering, (2) weight sum ordering, (3) unique value ordering, and (4) shortest path ordering [16]

This means that there are strongly connected components, and the nodes within them share similar characteristics. Users can also see that the components increase in size from the top-left to the bottom-right corner of the matrix, where the connected components are the largest. In addition, in each component, the edges are ordered by their weight from the bottom-right corner of the component to the top-left corner, where the weight of the edges is the largest. In Fig. 13, the adjacency matrix displays the same dataset using the mean value ordering (1), weight sum ordering (2), unique value ordering (3), and shortest path ordering (4).

Users can see the reordered matrices in (1), (2), and (3) ordering the edges by weight from the top-left to the bottom-right corner of the matrix, where the weight of the edges is the largest. The reordered matrices (3) and (4) form block patterns, which also confirm that there are strongly connected components in the data.

The node-link layouts as well as the matrix reordering can be applied to all graphs in a graph sequence or even individual graphs. Selecting node groups individually following certain visual properties can help visually identify the selected node groups in other diagrams, or applying algorithmic approaches can automatically detect commonalities of selected node groups, for example, their overlap. Based on these visual and algorithm identification processes, hints about strongly connected nodes can be obtained whose connection is based on several visual outcomes, not an individual one, as in standard graph visualization techniques. In particular, for dynamic graphs, such a multiple perspective identification feature might be useful for understanding stable and unstable node groups in graph evolution. The usefulness of the technique can be shown best if several graphs are under exploration, for example, a sequence of static graphs, that is, a dynamic graph. An example scenario for such a case is described in the following section.

### Exploring dynamic graphs

The co-authorship graph persists over a longer time span, one for each year, which makes the static graph change dynamically. Exploring groups or clusters of co-authors over the years and how the core of the cluster has evolved over time is a tedious task, particularly if the graph data have evolved over several decades. Although inspecting a side-by-side visualization of the graph sequence can give a first impression of the data, understanding the evolution of clusters remains a challenging task if the core of the cluster changes dynamically.

**Fig. 14:**
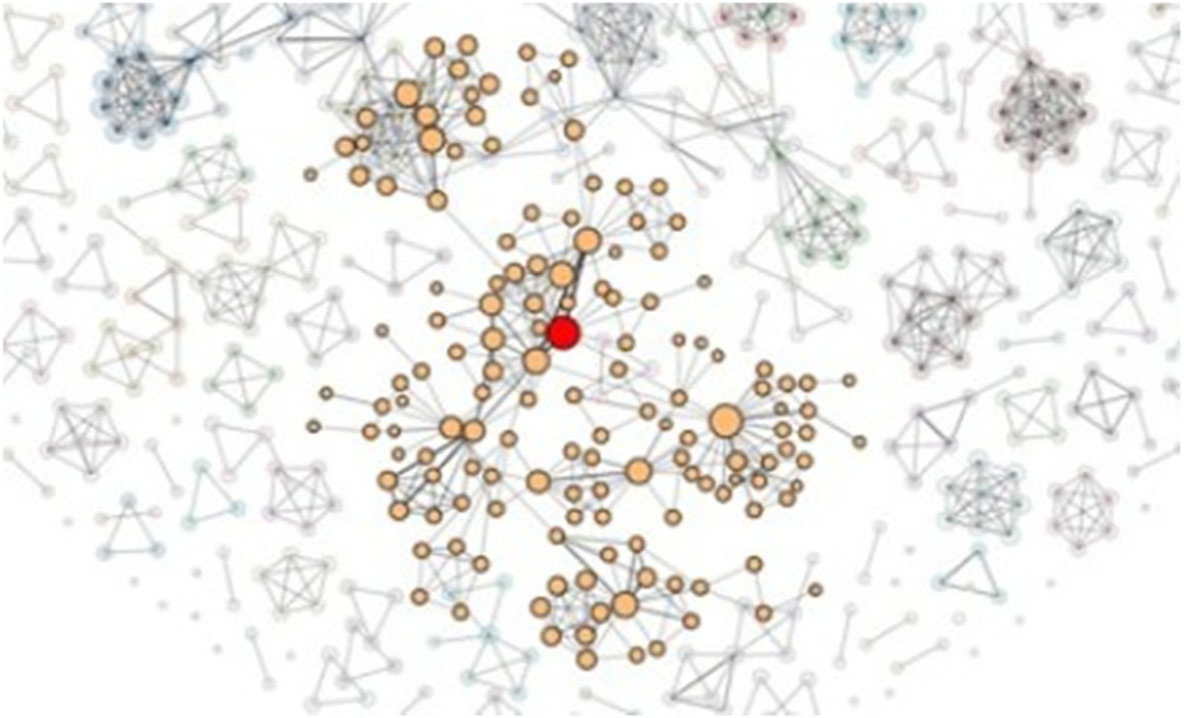
Selecting a node representing the author Jock Mackinlay while applying a cluster detection algorithm based on nearest neighbors in the graph to compute a group of co-authors. It should be noted that, although this subgraph might be selected manually, it might not be exact, and a mixture of algorithmic and manual solutions is also possible

Hence, it might be a wise decision to only visually observe one static graph from the graph sequence (maybe the latest one), select an interesting node, compute a corresponding cluster in the static graph (Fig. 14), and apply an intersection algorithm that computes the stable parts of this cluster over time (to see which co-authors conducted research together over longer time spans).

However, simply selecting groups of nodes visually might not be as exact as the use of algorithmic cluster detection algorithms, particularly for all time steps in the graph sequence. Moreover, a visual inspection of a multitude of graphs is a tedious and time-consuming task, whereas a pure algorithmic selection does not have such an effect, as shown by visual patterns or outliers that can only be detected by human users owing to human perceptual capabilities. Consequently, a mixture based on user decisions, selections, and algorithmic solutions can be the key to success under this situation.

**Fig. 15:**
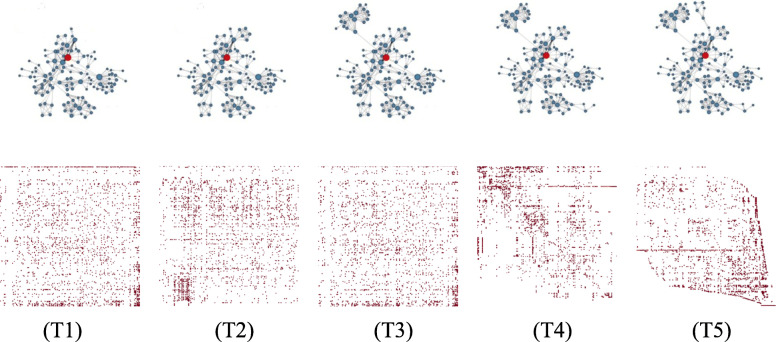
Inspecting the dynamics of the selected group of co-authors of Jock Mackinlay from Fig. 14 in a force-directed node-link diagram indicates the growth of the co-author network over time, from (T1) to (T5) (top row). In a corresponding adjacency matrix in different reordering strategies, the group building can be further refined in a visual way (bottom row). However, in the matrix example here, it is quite difficult to select individual nodes owing to the original dataset size. The selected co-author group in the node-link diagram was based on time step (T5)

As an add-on of the present study, a group of nodes can be visually selected and their evolution over time can be shown, not only in a single dynamic graph visualization, but also in several visualizations, based on different node-link layouts or an adjacency matrix reordering of the individual static graphs (see Fig. 15 for a sequence of force-directed node-link diagrams for a pre-selected cluster from a larger co-authorship graph and adjacency matrices for each time step, and for illustrative purposes, in different matrix reorderings for the entire graph). With this concept, it helps to start with a user-selected number of nodes, while comparing them in different views to identify the most connected nodes. A pure algorithmic solution might not lead to the best option because several relevant nodes might be missed by the algorithm because of the missing semantic information as well as an understanding of the algorithm for the visual patterns in a node-link diagram or adjacency matrix. Human users are faster and more accurate when identifying visual patterns, particularly if many layouts and a matrix-reordering are used; however, a clever combination with an algorithmic approach can generate an even more suitable solution.

## User study

A small user experiment was conducted by recruiting 15 students from a visualization course. The students had prior knowledge of graph visualization from lectures and assignments during the course. The ages of the students ranged from 21 to 29 years, with an average age of 23.4 years. The student population consisted of 10 men and 5 women. Seven of them wore glasses or contact lenses.

### Study procedure

The students were given textual instructions by reading a description of the tool. After stating that they understood the tool, they were given a tool demo by using the experimenter’s laptop with the tool pre-installed. The students were allowed to practice using with the interactive features until they felt comfortable. The experimenter asked some test questions to check if they were able to work with the tool. Moreover, although they had to fill in their personal details on a form, their names were not requested for privacy reasons, i.e., the recorded data were anonymized.

### User tasks

The first task (T1) for the students was to familiarize themselves with the visualization tool. As a second, but major task (T2), they were shown a node group consisting of 10 nodes in a node-link diagram depicted in a force-directed layout. The corresponding 10 nodes are highlighted in red to make them pre-attentively detectable. They were instructed to use the proposed interactive visualization tool to describe in which other node-link layouts and adjacency matrix reordering the given node group behaves similarly, that is, if they form a cluster or a certain type of flow in a hierarchical layout. As an add-on task (T3), they were instructed to report whether the selected node group persisted over a longer time period and in which layout or matrix the reordering style could most easily be identified.

## Results

The results are based on three tasks and qualitative feedback provided by the students.

Task T1: After 8 min and 13 s on average, they reported familiarity with the tool, with the fastest student requiring 5 min and 17 s and the slowest 17 min and 7 s.

Task T2: They identified the hierarchical layout as less useful for this task, but found the node group in the circular layout to be the most visually effective. It took 1 min and 53 s on an average to solve this task.

Task T3: All students determined that the node group exists over 11 time steps and then quickly decreases in size for 3 time steps, returning again for 5 more time steps before disappearing permanently. The best node-link layout for this task was the force-directed layout, which was Cuthill–McKee ordering because the nodes in the group were placed close together and appeared as a common cluster. The average response time for this task was 2 min and 12 s.

### Qualitative feedback

Apart from the task solutions, user opinions in the form of qualitative feedback are also of interest. Moreover, the students were instructed to think and talk aloud during the running experiments. The experimenter noted the verbal remarks and included them in the summary.

In particular, some students reported that they wished to have more views placed next to each other, instead of having just two to see the layouts and matrix arrangements side-by-side. They also asked for more interaction techniques, particularly for zooming in and out as well as overview-and-detail or focus-and-context techniques. Moreover, further label-based selection processes are required to better guide the exploration process, and it is difficult to select individual pixels in the adjacency matrix visualization. They also asked for a search function for nodes based on certain node properties or even subcommunities, such as nearest neighbors in the graph. Finally, a history function used to look back during the exploration process was considered; however, this is a challenging feature to implement.

## Conclusion and future work

In its current state, the proposed web-based tool has achieved its goal of rendering static and dynamic graph visualizations, which can be used to detect patterns and insights from the graph data by combining several layouts and reorderings. The tool provides useful layouts, interactions, and reordering strategies that help the user achieve this. However, there are always aspects that can be improved to make the proposed application even better. Currently, the tool can only work with datasets in a specific format, which is not user-friendly. To improve this, the proposed application should support all possible datasets or at least the most common ones. Although the proposed tool works on touchscreen devices, there are issues related to panning, zooming, and selecting elements. It would also be useful to extend the repertoire of visual feature combinations, for example, by considering further node and edge properties as well as the topology of the underlying graph structure. Moreover, an even more thorough user study must be conducted to understand the real potential or design flaws of the tool. Eye tracking is a powerful technology for finding insight into the visual attention patterns of human users.

## Data Availability

Not applicable.

## Abbreviations

CPAN: Comprehensible Perl Archive Network
SVG: Scalable vector graphics
D3: JavaScript-based visualization library
